# Auditory Brainstem Response Findings in Children with Level 1 Autism Spectrum Disorder: A Comparative Study

**DOI:** 10.1055/s-0044-1792084

**Published:** 2025-05-12

**Authors:** Mariana de Medeiros Cardoso, Rudimar dos Santos Riesgo, Pricila Sleifer

**Affiliations:** 1 Graduate Program in Child and Adolescent Health, Universidade Federal do Rio Grande do Sul (UFRGS), Porto Alegre, RS, Brazil; 2 Program in Child and Adolescent Health, Universidade Federal do Rio Grande do Sul (UFRGS), Porto Alegre, RS, Brazil; 3 Department of Health and Human Communication, Universidade Federal do Rio Grande do Sul (UFRGS), Porto Alegre, RS, Brazil

**Keywords:** electrophysiology, evoked potentials, auditory, brain stem, child, autism, autism spectrum disorders

## Abstract

**Introduction** Autism spectrum disorder is a pervasive developmental disorder characterized by deficits in communication and social interactions, as well as repetitive behavioral patterns. Understanding the relationship between auditory brainstem response and hearing is crucial, considering the importance of sensory function. Auditory brainstem response testing is a tool that evaluates the auditory system from periphery to brainstem in response to an acoustic stimulus, providing important information about the auditory pathways.

**Objective** To compare auditory brainstem response findings in children with autism spectrum disorder versus those of a control group.

**Methods** Cross-sectional, comparative study of 23 children (age 7–10 years) diagnosed with autism spectrum disorder and an age- and sex-matched control group of normal-hearing children with typical development. All participants underwent otoscopy, impedance audiometry, pure-tone audiometry, speech audiometry, and brainstem evoked response audiometry.

**Results** Statistically significant between-group differences were seen on comparison of the absolute latencies of waves III ( *p* = 0.047) and V ( *p* = 0.034), as well as interpeak intervals III to V ( *p* = 0.048) and I to V ( *p* = 0.036), with increased values in the study group. The sample was composed of 8.7% females and 91.3% males.

**Conclusion** In this sample, children with autism spectrum disorder showed increased auditory brainstem response latencies compared to the control group, suggesting auditory pathway impairment.

## Introduction

Autism spectrum disorder (ASD), formerly known as pervasive developmental disorder, is characterized by atypical development manifesting in the first years of life. 1 Deficits in communication and social interaction, as well as restrictive, repetitive, and/or stereotyped behavioral patterns, are among the most common manifestations. 2 Diagnosis is essentially clinical, based on direct observation, interviews with parents and/or caregivers, and the administration of specific instruments. 3 Children with ASD require a multifactorial, multidisciplinary treatment approach. 4

In recent years, there has been an exponential rise in ASD diagnoses. The worldwide prevalence is now estimated at 1 to 2 cases per 100 individuals. 5 In a more recent survey carried out with more than 226,000 children at the age of 8 years, the United States Centers for Disease Control and Prevention (CDC) demonstrated an even higher prevalence of ASD in the country—1 in 36, which amounts to 2.8% of the population—and reported that ASD was 4.3 times more common among boys than among girls. 6 These figures represent a 22% rise from the previous survey, which found a prevalence of 1 in 44. 5

Auditory issues, such as auditory sensitivity, are quite common and can trigger somatic symptoms. 6 7 8 People with ASD may also present with hyperacusis and abnormal central auditory processing. 9 Consequently, hearing-related disorders must be considered and appropriately diagnosed by means of a thorough and careful assessment. 10 This assessment requires special attention to potential challenges in communication and social interaction, considering that sensory changes in this population are common and can be confused with hearing impairment. 11

The integrity of the auditory pathway can be assessed through auditory brainstem response (ABR) testing (also known as brainstem evoked response audiometry, BERA, or brainstem auditory evoked potentials, BAEPs), which traces the signal conveyed from the auditory nerve to the brainstem. 12 Auditory brainstem response testing is a useful procedure in neonatal screening and in the workup of individuals of any age with neurological disorders, including ASD, as measurements are obtained objectively, without requiring active patient participation. 13

Several studies have addressed this topic, demonstrating a wide range of ABR findings in individuals with ASD. Some studies 14 15 16 17 18 19 20 21 22 23 24 reported an increase in wave-III latency, suggesting potential dysfunction of the cochlear nucleus. Others 16 17 18 20 21 22 24 25 26 27 28 reported increased wave-V latency, suggesting potential changes in functioning of the lateral lemniscus. 29 Additional reports 14 16 18 21 24 25 26 27 30 31 32 33 have noted increased I-to-III and/or III-to-V interpeak latencies. Taken together, these findings suggest that children with ASD may exhibit changes in neural synchrony and abnormalities at the level of the brainstem.

Many studies, 13 34 35 36 37 however, have found otherwise. When investigating absolute latencies, interpeak latencies, and amplitudes of ABR waves, these studies did not find statistically significant differences between age groups, which indicates integrity of the auditory pathway regardless of age in the context of ASD.

The importance of ABR findings in children diagnosed with ASD justifies further investigation, both to contribute to future studies and clinical practice and to expand the evidence base on this topic. Within this context, our primary objective was to analyze and compare ABR findings obtained in children with ASD to those obtained in a typically developing control group. As specific objectives, we tested for and compared the presence of correlations of the results obtained with age and sex between the study and control groups.

## Methods

This prospective, cross-sectional, comparative study was approved by the institution's Research Ethics Committee with opinion number 7900517.2.5334. Parents or guardians, as well as children, received guidance on the study procedures. All who agreed to participate assented to the evaluations being conducted and their guardians provided written consent to the storage of data and its subsequent use in scientific publications.

A non-probability convenience sampling approach was used. The sample was composed of children of both sexes, aged 7 years to 10 years 11 months, divided into 2 groups: study (SG) and control (CG). The SG was composed of children diagnosed with ASD and recruited from the outpatient ASD clinic of a public referral hospital. The CG was composed of normal-hearing students recruited from public schools affiliated with an extension project implemented and conducted by our institution, under the coordination of the supervisor of the present study.

The inclusion criteria were previous diagnosis of ASD by a pediatric neurologist (for the SG) and typical neurodevelopment (for the CG). The following inclusion criteria applied to both groups (SG and CG): absence of cerumen impaction at otoscopy; hearing thresholds within normal limits 38 bilaterally; a type-A tympanogram 39 and presence of acoustic reflexes at normal levels; and absence of ear disease (recurrent ear infections, tinnitus, other diagnosis of auditory system dysfunction). Children with intellectual disabilities, psychiatric and/or neurological disorders, genetic syndromes, craniofacial anomalies, or any other illness reported by the child's parents or guardians or confirmed by medical diagnosis, as well as those who did not understand or were unable for any reason to perform or complete the tasks and assessments stipulated in the study protocol, were excluded from the sample.

These procedures included a thorough history-taking, inspection of the external auditory canals, impedance audiometry to obtain acoustic immittance measures (AIM), pure-tone audiometry (PTA), speech audiometry, and ABR testing. To avoid error or misunderstanding of instructions, guidance was provided to participants in advance of the procedures; parents or guardians and the children themselves were informed of what would happen at each stage of the assessment before any procedures were performed.

Assessment began with a thorough history taking, in the presence of the child's parents or guardians, with the aim of collecting relevant personal information and information about the child's development, especially in relation to potential auditory issues, such as hearing history, hearing-related complaints, history of otitis, and hypo- or hypersensitivity to sound, among others. Visual inspection of the external auditory canals was then performed using a Welch Allyn otoscope (Welch Allyn, Inc., Auburn, NY, USA) to confirm eligibility for subsequent audiological testing.

Acoustic immittance measurements were obtained with an Interacoustics AT235h tympanometer (Interacoustics, Middelfart, Denmark). Static and dynamic compliance were evaluated and classified as reported elsewhere. 39 Acoustic reflex thresholds, both ipsilateral and contralateral, at frequencies from 500 to 4,000 Hz, were also investigated.

Pure-tone audiometry was performed in an acoustically treated booth using an Inventis Harp audiometer (Inventis, Padova, Italy) and calibrated supra-aural headphones. Air-conduction thresholds were obtained at 250 to 8,000 Hz and bone-conduction thresholds at 500 to 4,000 Hz, in both ears. Any detected hearing losses were diagnosed in accordance with the World Health Organization (WHO) classification. 38 Speech audiometry consisted of determination of the speech reception threshold (SRT), that is, the minimum hearing level at which the patient is able to understand 50% of a set of 3-syllable words presented, and the word recognition score (WRS), which involves presentation of 25 single-syllable words at a hearing level 40 dB above the 3-frequency pure-tone average (500, 1,000, and 2,000 Hz) and calculation of the percentage of words correctly repeated. For participants who were unable to complete speech audiometry due to inability to repeat words, pictures were presented instead, and the participant was asked to point to the picture corresponding to the presented word.

At a second encounter, after confirmation of the tympanometric curve and, thus, of middle-ear integrity and function, ABR testing was performed using a Contronic Masbe ATC Plus system (Contronic, Pelotas, RS, Brazil). The children were placed on a recliner to ensure comfort during the procedure. Using gauze pads, the examiner applied abrasive skin prep gel (NuPrep - Weaver and Company, Aurora, CO, USA) to the electrode placement sites. Silver electrodes were positioned on the forehead (Fz, active electrode; Fpz, ground electrode) and mastoids (reference electrodes: M1 for the left ear and M2 for the right ear). E-A-RTONE GOLD insert earphones (3M Company, Saint Paul, MN, USA) were placed in both ears. Electrical impedance was kept below 5Ω in each lead, and the difference between the 3 electrodes did not exceed 2Ω.

Once impedance had been confirmed, an electroencephalogram (EEG) was performed to capture any spontaneous cerebral electrical activity, checking for artifacts that could interfere with the test. All data collection took place while participants were in natural sleep. A click stimulus was used at an intensity of 80 dBnHL in the right and left ears separately (monaurally), in rarefaction polarity, at a rate of 27.7 stimuli per second. Each scan consisted of 2,048 stimuli, with a recording window of 12 ms, using 100-to-3,000 Hz filters and a rejection level of up to 10% of the total stimuli presented. To ensure waveform reproducibility, at least two tracings were obtained in each ear.

The latency and amplitude values of waves I, III, and V, as well as the I-to-III, III-to-V, and I-to-V interpeaks, were analyzed. To ensure greater reliability, the tracings were analyzed independently by two masked, experienced electrophysiologists at different time points. The findings were considered valid only when there was agreement between the evaluators' scores.

After collection, data were organized into descriptive statistics. Quantitative variables were expressed as means, standard deviations, and ranges, while qualitative variables were described as absolute and relative frequencies. A significance level of 5% ( *p* < 0.05) and 95% confidence intervals (CIs) were used. All analyses were conducted in the IBM SPSS Statistics for Windows, version 21.0 (IBM CORP., Armonk, NY USA). Inter-rater agreement in ABR analysis was evaluated using the Kappa statistic, interpreted as described elsewhere. 40 Correlation was classified as poor, negligible, weak, moderate, substantial, or almost perfect based on Kappa values. The intraclass correlation coefficient (I) was interpreted according to the Fleiss classification, 41 and categorized as poor, satisfactory, or excellent in terms of correlation strength.

## Results

A total of 46 children aged 7 to 10 years (mean: 8.3 ± 1.5 years) participated in the study. Three children were excluded from the sample as they were unable to complete all proposed assessments. The results presented below correspond to a sample of 23 children in the study group (SG), 2 females (8.7%) and 21 males (91.3%), and 23 age- and sex-matched children in the control group (CG). All excluded participants received appropriate guidance and referrals.

No statistically significant differences were observed between the right and left ears within either group ( Table 1 ); therefore, values were pooled by ear and compared between groups. The absolute latency values of waves I, III, and V and the interpeak intervals I to III, III to V, and I to V are given for the study and control groups.

**Table 1:** Auditory brainstem response findings in children diagnosed with autism spectrum disorder (study group) and typically developing peers (control group)

BAEP Latency (ms)	Study group	Control group	*p* *
	(n = 23)	(n = 23)	
	Mean ± SD	Mean ± SD	
Wave I	1.55 ± 0.20	1.50 ± 0.15	0.125
Wave III	3.85 ± 0.25	3.60 ± 0.20	0.047
Wave V	5.85 ± 0.25	5.65 ± 0.25	0.034
I–III interval	2.25 ± 0.25	2.20 ± 0.25	0.097
III–V interval	2.20 ± 0.20	2.00 ± 0.15	0.048
I–V interval	4.35 ± 0.15	4.00 ± 0.20	0.036

Abbreviations: BAEP, brainstem auditory evoked potentials; control group, age- and sex-matched neurotypical children; ms, millisecond; n, number of subjects in the sample; SD, standard deviation; Study group, children with autism spectrum disorder.

Note: *Student *t* -test, significant at *p* < 0.05.

There was a statistically significant difference in absolute latency values for waves III ( *p* = 0.047) and V ( *p* = 0.034) as well as the III-to-V ( *p* = 0.048) and I-to-V ( *p* = 0.036) interpeak intervals between groups, with the study group (children with ASD) showing increased latencies compared to the control group.

All SG children had been diagnosed with level 1 ASD (requires support for activities of daily living). 2 Furthermore, according to information provided during the history, all parents or guardians stated that the children had auditory hypersensitivity.

On qualitative evaluation of the waveforms, three boys from the study group exhibited altered morphology, with adequate wave reproducibility and wave-I amplitude similar to the amplitude of wave V.

## Discussion

Research on ABR in children with ASD has yet to yield consensual findings. However, several studies have demonstrated altered latency values. 14 15 16 17 18 20 21 22 23 24 25 26 27 30 31 32 33 42 43 44 45 46

The male predominance (91.3%) identified in this study is consistent with the overall scientific literature on children with ASD. 16 21 35 47 The prevalence of ASD is significantly higher in males than in females; Maenner et al *.* 5 report that it is 4.3 times more common in males (1 in every 34 boys) than in females (1 in every 145 girls). The reasons for this gender disparity in the prevalence of ASD are not yet completely understood. Some investigators have suggested that genetic, hormonal, neurological, and environmental factors may all play a role in this difference. 9 48 49 Loomes et al *.* 50 conducted a systematic review of 54 studies including a total of 53,712 participants diagnosed with ASD and found that females may simply be underdiagnosed instead, as they may have more developed social skills, thus masking some symptoms of ASD. They also note the potential for gender bias at the time of diagnosis, meaning that some girls are at high risk of not receiving an appropriate diagnosis even if they meet criteria for ASD.

We found no statistically significant differences in BAEP findings between the right and left ears in either group; this is consistent with the existing literature. 13 18 21 31 We did, however, find statistically significant increases in the latency of waves III and V and interpeak intervals III-to-V and I-to-V in the study (ASD) group compared to the typically developing control group. These differences suggest that children with ASD may have abnormal conduction of neural impulses between the cochlear nucleus (wave-III generating site) and the lateral lemniscus (wave-V generating site), denoting changes in neural synchrony. 29 51 52 This, in turn, is indicative of potential dysfunctions in the central auditory nervous system (CANS) at the level of the brainstem, as changes in BAEP absolute latencies, interpeak intervals, and wave amplitudes may be associated with abnormalities of myelination, axon diameter, or synaptic efficacy of the brainstem auditory pathways. 28 53

Our findings highlight the complexity of the auditory system and corroborate the results of previous studies. 14 15 16 17 18 20 21 22 23 24 26 27 28 42 45

Increased absolute wave-V latency is one of the most frequent findings described in the scientific literature on ABR. 14 16 17 18 20 21 22 Increases in wave-V latency may be associated with altered neural conduction along the auditory pathway, thus affecting the speed of information processing. 18 21 24 27

In individuals with ASD, the integration of auditory stimuli may be compromised, resulting in difficulties in interpreting and responding to sounds. 28 These issues can range from increased sensitivity to certain sounds (hyperacusis) to difficulty understanding complex sound patterns. 16 54 Miron et al. 18 stress that prolongation of wave V is not a finding exclusive to ASD, but can also be found in children with language delay. It is worth noting that ASD is associated with challenges in sensory processing, including integration of auditory stimuli.

Changes in interpeak intervals I to V and III to V have been described in previous studies. 14 16 18 21 24 25 26 27 30 31 32 33 Interpeak intervals I to V and III to V represent the time difference between the different ABR waves and suggest altered conduction of electrical impulses along the auditory pathways. 12 23 In children with ASD, prolongation of these intervals may indicate a delay in information processing within the auditory pathways. This may be associated with delays in the actual transmission of sound stimuli or challenges involving neural processing. 14 16 21

It has been inferred that altered wave latencies suggest possible delays or dysfunctions in the maturation of the central auditory pathway in children with ASD. 18 21 23 It is important to consider that maturation of the auditory system is a complex process which can be affected by several variables, including genetic and environmental factors. 12 53 55

Auditory hypersensitivity was reported by the parents and/or caregivers of all children in the study group during the initial history. Sensory disorders affect up to 90% of individuals with ASD, with auditory sensitivity being more common than visual or proprioceptive sensitivity. 1 7 8 10 26 56 A study carried out on children and adolescents with ASD reported an 88.2% prevalence of auditory hypersensitivity and found that these sensory issues can contribute to changes in central auditory processing abilities. The auditory sensitivity experienced by this population may also be associated with prolonged wave-V latency, denoting impaired integration of auditory and visual information. 57

The pathophysiology of auditory hypersensitivity is still incompletely understood, regardless of the population. 10 However, some hypotheses can be considered: homeostatic plasticity of the CANS, which regulates the precision of neural coding 58 ; tonotopic organization in primary areas, resulting in overrepresentation of certain sounds 59 ; and failure to modulate the efferent fibers of the olivocochlear system, which regulate sound amplification. 54 60

It bears stressing that three male participants in the study group exhibited abnormal morphology of the ABR waveforms, but with adequate replicability, and wave-I amplitude similar to wave-V amplitude, that is, increased. This finding corroborates previous studies, which found a wave I-amplitude greater than wave-V amplitude in individuals with ASD and is indicative of abnormal peripheral auditory system function and/or the presence of other cerebral anomalies that influence the efferent auditory system and may contribute to hyperacusis. 15 43 44 45 46 Several authors 16 18 20 25 32 have further inferred that subjects with ASD may exhibit lower amplitudes and greater delays in brainstem response.

In typically developing young children, the wave-I amplitude can be equal to or, in some cases, even greater than the amplitude of wave V. 29 55 However, this amplitude tends to decrease as the auditory pathway matures, and approximately around 12 and 18 months of age, values are already similar to those observed in adults. 4 55 Therefore, we expected, that in the population analyzed in this study, the amplitude of wave I would be smaller than that of wave V. It is noteworthy that wave amplitude results from the synchrony of the auditory nerve action potential and, consequently, the number of neural fibers activated during exposure to the sound stimulus. 61

The visual pattern of ABR waves, known as the ABR morphology, demonstrates neuronal involvement and the response to auditory stimuli. 62 Central auditory nervous system maturation is a crucial element in this analysis. 55 However, it should be noted that there is no standardized classification in the literature for analysis of BAEPs. Although wave morphology analysis revealed abnormalities in some of our SG participants, we cannot correlate these with wave amplitude values. This finding demonstrates the heterogeneity and variability of the manifestations of ASD.

It is worth highlighting that several previous studies have failed to find any statistically significant differences in absolute latencies, interpeak latencies, or ABR amplitudes, thus suggesting integrity of the auditory pathways, in populations with ASD. 13 34 35 36 37

This wide variety of changes described in the literature on ABR in individuals with ASD may be associated with the heterogeneity of clinical characteristics in this population, as noted above. 63 The diagnosis of ASD has undergone a significant perspective shift; once previously applied mainly to children with severely atypical development, it now includes different levels of support requirements. 2 64 It can be inferred that, due to this heterogeneity of manifestations, the results of ABR testing with click stimuli may vary widely depending on the tested sample, and this must be taken into account in the discussion of these findings. 31 47 It is essential to recognize that ASD is not a single diagnosis, but rather, as its name implies, a spectrum covering different levels of severity. 64 This characteristic heterogeneity of ASD allows classification into subgroups. ABR latency changes may occur in certain subgroups, but not in others.

The present study reports findings relevant for clinical practice and should encourage future research, as it demonstrates that ABR testing can assist in the assessment process and enable earlier therapeutic intervention. One limitation is that the sample of this study was restricted in age range (7–10 years) and severity of ASD (all participants were level 1). Therefore, our findings cannot be generalized. Additional research is needed to improve our understanding of the auditory brainstem responses of children with ASD. Such research should provide essential inputs to inform the evaluation and, consequently, treatment and follow-up of this population.

## Conclusion

The findings of the present study demonstrate that children with ASD, regardless of age or sex, may exhibit abnormal ABR when compared to typically developing children. In our sample, there were statistically significant differences in the absolute latency of waves III and V, as well as in III-to-V and I-to-V interpeak latencies, between the control and ASD groups. These findings may denote a change in sound conduction at the brainstem level.

Analysis of ABR in children with ASD can contribute to a better understanding of neuroelectric conduction in the auditory pathway and thus help guide increasingly early interventions, as well as future research aiming at a more comprehensive characterization of these electrophysiological phenomena. Additional studies are needed to better understand the findings obtained in this population.
